# Influence of host phylogeny, geographical location and seed harvesting diet on the bacterial community of globally distributed* Pheidole* ants

**DOI:** 10.7717/peerj.8492

**Published:** 2020-02-04

**Authors:** Cíntia Martins, Corrie S. Moreau

**Affiliations:** 1 Department of Biological Science, Campus Ministro Reis Velloso, Universidade Federal do Piauí, Parnaíba, Piauí, Brazil; 2 Department of Science and Education, Field Museum of Natural History, Chicago, IL, United States of America; 3 Departments of Entomology and Ecology & Evolutionary Biology, Cornell University, Ithaca, NY, United States of America

**Keywords:** Granivory, NGS, Microbiota, Formicidae, Myrmicinae

## Abstract

The presence of symbiotic relationships between organisms is a common phenomenon found across the tree of life. In particular, the association of bacterial symbionts with ants is an active area of study. This close relationship between ants and microbes can significantly impact host biology and is also considered one of the driving forces in ant evolution and diversification. Diet flexibility of ants may explain the evolutionary success of the group, which may be achieved by the presence of endosymbionts that aid in nutrition acquisition from a variety of food sources. With more than 1,140 species, ants from the genus *Pheidole* have a worldwide distribution and an important role in harvesting seeds; this behavior is believed to be a possible key innovation leading to the diversification of this group. This is the first study to investigate the bacterial community associated with *Pheidole* using next generation sequencing (NGS) to explore the influences of host phylogeny, geographic location and food preference in shaping the microbial community. In addition, we explore if there are any microbiota signatures related to granivory. We identified Proteobacteria and Firmicutes as the major phyla associated with these ants. The core microbiome in *Pheidole* (those found in >50% of all samples) was composed of 14 ASVs and the most prevalent are family Burkholderiaceae and the genera *Acinetobacter*, *Streptococcus*, *Staphylococcus*, *Cloacibacterium* and *Ralstonia*. We found that geographical location and food resource may influence the bacterial community of *Pheidole* ants. These results demonstrate that *Pheidole* has a relatively stable microbiota across species, which suggests the bacterial community may serve a generalized function in this group.

## Introduction

Symbiotic bacteria in insects have been the focus of several studies in the past few years and the number of studies has increased since next generation sequencing methods have continued to advance (Feldhaar, 2011; Russell, Sanders & Moreau, 2017). The impacts symbiotic associations have on the species involved have been recognized as an important driver of evolution (Anderson et al., 2012; Bennett & Moran, 2015). Insects, with special emphasis on ants, are known to depend on symbionts for a number of reasons including dietary dependence for food processing and nitrogen or vitamin enrichment (Feldhaar et al., 2007; Feldhaar, 2011; Hu et al., 2018).

Ants are a highly diverse group of insects distributed in most terrestrial environments with more than 13,000 species described (Bolton, 2018) and are a group that has been the focus of many studies related to symbiotic bacteria due to their great ecological success and high species diversity. Recently, several studies have focused on microbial interactions with ants but we are just starting to understand how the ecology and evolution of ants and bacterial symbionts has been shaped over time (Russell, Sanders & Moreau, 2017). In several recent studies, certain ant species have been associated with low bacteria densities (i.e., *Crematogaster* from Rubin et al., 2014, *Solenopsis* from Ishak et al., 2011, *Linepithema* from Hu et al., 2017, and several additional ant genera from Sanders et al., 2017a; Sanders et al., 2017b), while others are associated with high bacteria diversity (*Cephalotes* from Hu et al. (2014), Lanan et al. (2016); and several ant genera from (Sanders et al., 2017a; Sanders et al., 2017b) with special attention given to herbivorous ants that harbor a nutritional mutualism with symbionts providing essential nutrients by several pathways (Hu et al., 2018). However, some ant groups with other diets, such as seed harvest, that could be related to symbiont association have not been studied in detail.

To address this question, we studied the hyper-diverse and worldwide distributed *Pheidole* ants. Recognized as one of the most species-rich genera with more than 1,140 species described worldwide (Bolton, 2018; Wilson, 2003), it has been suggested that this clade first evolved in the New World with one introduction into the Old Word (Moreau, 2008; Economo et al., 2015). *Pheidole* ants have the fascinating potentially key innovation of seed milling and harvesting; an innovation that has great importance in driving plant diversity by dispersal of seed (myrmecochory) (Lengyel et al., 2009). This diet preference may have been lost or emerged multiple times throughout the evolution of *Pheidole*. This unique behavior is suggested as one of the causes of the high success and diversification of this group (Moreau, 2008). Because some *Pheidole* ants feed on plant seeds, they can incidentally aid in plant dispersal (Thomson et al., 2016) and the rise of angiosperms is one important factor that may have led to ant diversification (Moreau et al., 2006) highlighting the importance of flowering plants to this group of insects. Despite the importance in the evolution and diversification of plants and their symbiotic ant partners, little is known about the forces shaping granivory as a new food resource in ants and, to date, no study has investigated the microbiota associated with *Pheidole* using next generation sequencing. Therefore, one of the main questions of our study is if there are signatures of microorganisms helping these ants to leverage this novel food resource.

Only a small number of insects with seed harvesting habits have had their microbiome evaluated. Two studies have shown interesting results related to one specific Betaproteobacteria genus, *Ralstonia*. In Carabid beetles (which consume insect prey as well as seeds), these bacteria were found associated with dietary treatment of seeds (Lundgren & Lehman, 2010). In a study to determine the bacterial symbionts associated with the seed-parasitic insect *Megastigmus* (Hymenoptera: Torymidae) Paulson, Von Aderkas & Perlman (2014) also found *Ralstonia* as the dominant bacteria genus. In a subsequent study of *Megastigmus* with transcriptome analysis, many mobile genetic elements transcripts from *Ralstonia* were discovered (Paulson et al., 2016), corroborating the association of this bacterial genus with *Megastigmus*. In spite of these results, no function of *Ralstonia* was determined in these insects and a gap still remains in the seed feeding insect microbiome which requires further study.

Despite the ecological importance of *Pheidole*, and its high diversity and wide distribution, little is known about the evolutionary forces that drove its diversification and what potential role host-associated bacteria have in this group. Furthermore, symbionts associated with *Pheidole* species are not well known with the exception of *Wolbachia* (Russell et al., 2009a) and the description of associated *Rhizobiales* (Russell et al., 2009b). We used 16S rRNA gene amplicon sequencing to investigate the bacterial community diversity of *Pheidole* to better understand the diversification of *Pheidole* ants and how its microbiome may be related to their seed harvesting behavior. For this: (i) we investigated the main microbial community members associated with *Pheidole* ants; (ii) we analyzed how geography influences the bacterial community; (iii) we investigated if bacterial symbionts may explain the ability of many species of *Pheidole* to leverage new food resources such as seed harvesting; and (iv) we combined the bacterial community information with the *Pheidole* phylogeny from Moreau (2008) to further investigate the influences of the bacterial community in the evolution of this group of ants. Our results show that *Pheidole* has a core microbiome, with geographical location and food resource play an important role in shaping the bacterial community.

## Materials & Methods

For this study, we used the same *Pheidole* samples and DNA extractions as included in Moreau (2008), which represent a worldwide collection of *Pheidole* species. We carefully selected 118 samples from this study from over 100 unique species (Table S1) to cover the breadth of localities and diets encompassed in the genus. The samples were preserved in 95% ethanol and stored at −20 °C before DNA extraction. The taxonomic identification of ants followed Bolton (2003) and Wilson (2003). Entire individuals had total DNA extracted, and in those species with small workers two individuals were combined. The Qiagen DNeasy Tissue kit was used for DNA extraction as in the described protocol of Moreau (2014). The DNA extractions were implemented without modification of the Qiagen DNeasy kit for Gram-positive bacteria following the findings of Rubin et al. (2014). Although this method is able to detect Gram positive bacteria, this could still influence the diversity of bacteria we are able to detect.

### Bacterial quantification

We used quantitative PCR (qPCR) to measure the amount of bacterial DNA in each sample for checking sequencing efficiency and also to test differential bacterial abundance between ant samples. We used universal 16S rRNA gene primers 515F and 806R (Caporaso et al., 2012), SsoAdvanced 2X SYBR Green Supermix (Bio-Rad) and 2 µL of DNA following initial denaturation at 95° C for 3 min and 40 cycles of 95° C for 10 s, 50° C for 10 s, 72° C for 30 s. All the qPCRs were run and analyzed in triplicate on a CFX Connect Real-Time System (Bio-Rad, Hercules, CA). Standard dilutions from *Escherichia coli* 16S rRNA gene amplicons were used to generate standard curves. We averaged the starting quantity (SQ) values of the triplicates of each sample and log10 transformed before implementing *t*-test and ANOVA on R version 3.4.2 (R Development Core Team, 2017) to check the differences in 16S rRNA gene copy number among the different categories of ant samples. A box plot graphic was also generated using software R.

### Amplification of 16S rRNA gene **andIlluminaMiSeq2000 sequencing**

Amplification of 16S rRNA genes and sequencing followed the protocols from the Earth Microbiome Project (EMP, http://www.earthmicrobiome.org/protocols-and-standards/) through the MiSeq2000 platform (Caporaso et al., 2012). Amplification of 16S rRNA gene was performed in triplicate using the primers 515F and 806R (Caporaso et al., 2012). For this step, we also included “blank samples” (*n* = 2) which were comprised of water instead of DNA, and all the required reagents. Each reaction had a final volume of 25 µL, containing 13 µL PCR grade H_2_O, 10 µL of 5 Primer Hot Master Mix, 0,5 µL of each primer (forward and reverse) and 1 µL of target DNA with the following thermocycler parameters: initial denaturation at 94° C for 3 min followed by 35 cycles of 94° C for 45 s, 50° C for 60 s, 72° C for 90 s, and final extension at 72° C for 10 min. The amplicons obtained average 300–350 bp, and quantification was performed using a Qubit fluometer (Invitrogen), followed by purification using the QIAquick PCR Purification Kit (Qiagen), all in accordance with the manufacturer’s recommendations. Subsequently, quantification was performed using the NanoDrop 2000 (Thermo Scientific) spectrophotometer to confirm that the sample pool had the 1.8-2 260/280 ratio recommended for Illumina sequencing.

The samples that amplified with good yield were sequenced using the MiSeq V3 kit according to the manufacturer’s protocol on the NGS Illumina MiSeq 2000 platform housed in the DNA laboratory at the Field Museum of Natural History.

### Bioinformatics **and Biostatistics**

Sequencing results were analyzed with the QIIME 2 software package version 2018.8 (https://qiime2.org) (Bolyen et al., 2019) and plugins associated with this version. Paired-end reads was demultiplexed using the demux plugin (https://github.com/qiime2/q2-demux) with the demux emp-paired command. Quality control, filtering chimeric sequences and feature table construction was done using the q2-dada2 plugin (Callahan et al., 2016) with trimming parameters based on the demux visualization. Taxonomy classification alignment with 99% similarity was done against the SILVA 132 database (Quast et al., 2013; Yilmaz et al., 2014) using a pretrained naive Bayes classifier and the ‘feature-classifier’ plugin (Bokulich et al., 2018) with the ‘classify-sklearn’.

We used the Decontam package 1.2.1 version (Davis et al., 2018) in R version 3.5.2 to filter contaminants based on ASVs (amplicon sequence variants) present in our two “blank samples” in order to reduce contaminants from laboratory or reagent source in our sample. For this, we imported into R the metadata text file, our ‘feature-table.biom’ file and ‘taxonomy.qza’ and joined these three archives into a phyloseq-class object with the phyloseq package 1.26.1 version (McMurdie & Holmes, 2013). Using the Decontam package we calculated Decontam scores using frequency, prevalence and combined methods and then plotted a histogram to visualize which method best filtered our samples. The bimodal expected representation which indicates a good model of decontamination as described in (Davis et al., 2018) was found for our samples using the prevalence method. Filtering contaminants was done using the *isContaminat* function with prevalence approach using a threshold P* of 0.5 (Davis et al., 2018).

The ‘taxonomy’ and ‘feature-table’ were imported back to QIIME2 to proceed with analysis. An additional filtering step was applied to filter out samples with a total frequency less than 6,000 reads. Furthermore, low abundance features (i.e., ASV with low total abundance) were filtered from our ‘feature-table’ using –p-min-frequency 100 and taxonomy based filtering was conducted for features that contain mitochondria, chloroplast or eukaryotes in their taxonomic annotation by –p-exclude mitochondria, chloroplast and eukaryotes.

The resulting filtered sequences were used to reconstruct a phylogenetic tree using ‘align-to-tree-mafft-fasttree’ pipeline from the q2-phylogeny plugin (https://github.com/qiime2/q2-phylogeny) with the resulting rooted phylogenetic tree used for further phylogenetic diversity metrics. Alpha and beta diversity analysis was calculated through q2-diversity plugin (https://github.com/qiime2/q2-diversity) using ‘core-metrics-phylogenetic’ method with a sampling depth (rarefaction) of 7,000. Associations between categorical metadata columns (type of food resource, geography related to region and Old World x New World samples) and alpha diversity data were calculated through ‘qiime diversity alpha-group-significance’ with Kruskal-Wallis pairwise test (Kruskal & Wallis, 1952) for Shannon’s diversity index, Observed Features, Faith’s Phylogenetic Diversity and Evenness. In order to test if the distances between the sample in each metadata group (type of food resource and geography) are more similar to each other than to any other groups we used ‘qiime diversity beta-group-significance’ plugin (Anderson, 2001) using the PERMANOVA method (default). Since most of our tests did not show significance between all samples for each category in the PERMANOVA method, we looked for pairwise test between the groups inside each category to see if signals were hidden (i.e., in food resource we compared the seed, no seed and no info group, in region between all the seven regions, and between Old World and New World).

To test if there are any differentially abundant taxa in our sample groups (ants from different food resource and geography) we used a statistical power analysis called ANCOM (analysis of composition of microbiomes). For this we used the ANCOM plugin (Mandal et al., 2015) with ‘qiime composition ancom’ command.

To access the ‘core’ (bacteria found in >50% and in >40% of our samples—see justification below) we used ‘qiime feature-table core-features’ plugin and the resulting core identified was filtered from the original table that was obtained after Decontam, low abundance and taxonomy-based filtering. The resulting 50% ‘core’ was analyzed for alpha group significance, beta group significance and ANCOM following the same procedure as with the full bacterial community data. Since we studied many different *Pheidole* species (over 100 species) we choose to be conservative with our cut off for ‘core’ because we are looking at a host group with over 50 million years of evolution (Moreau, 2008) and a high cut off for ‘core’ could prevent identifying the ancient relationships between the bacterial community and host. Furthermore, we looked at a 40% ‘core’ community in order to visualize *Wolbachia* distribution across samples because this endosymbiont was found in 45.3% of all samples from this study (Table S5) and is one of the most prevalent in insects and we wanted to illustrate and visualize its distribution across *Pheidole* samples.

PCoA implemented through the ‘qiime diversity core-metrics-phylogenetic’ which applies a collection of phylogenetic and non-phylogenetic diversity metrics to a feature table was used to generate weighted and unweighted Unifrac distance matrix used in downstream beta diversity and also PCoA matrix computed from unweighted and weighted Unifrac.

To illustrate Pheidole’s ‘core’ bacterial communities (‘core’ bacteria found in >50% and to illustrate the presence of *Wolbachia* bacteria in >40% of our samples) we generated a heatmap representation of the feature table generated in the ‘core’ analysis using ‘qiime feature-table heatmap’ plugin (Hunter, 2007) with Bray–Curtis statistics to generate a hierarchical clustering of the bacterial communities and the samples.

In order to test if the phylogeny and fine scale geography is influencing the bacterial communities in *Pheidole* ants we used a Mantel test implemented in QIIME2. For these correlation tests, we used the distance matrix of the *Pheidole* phylogeny from Moreau (2008), the weighted and unweighted Unifrac distance matrices generated in the beta-diversity analysis (the full bacterial community not core-filtered) and the latitude and longitude from the location of each collection sample. The distance matrix obtained from the Moreau (2008) phylogeny was edited and calculated in R using the ape package (Paradis, Claude & Strimmer, 2004). The distance matrix generated in R was imported to QIIME2 as a distance matrix for further analysis. The ‘qiime diversity mantel’ plugin (Mantel, 1967) was used with both weighted and unweighted distance matrix from beta-diversity analysis. The correlation was tested between the *Pheidole* phylogeny and the bacterial community, the geography and the bacterial community.

## Results

**Figure 1:**
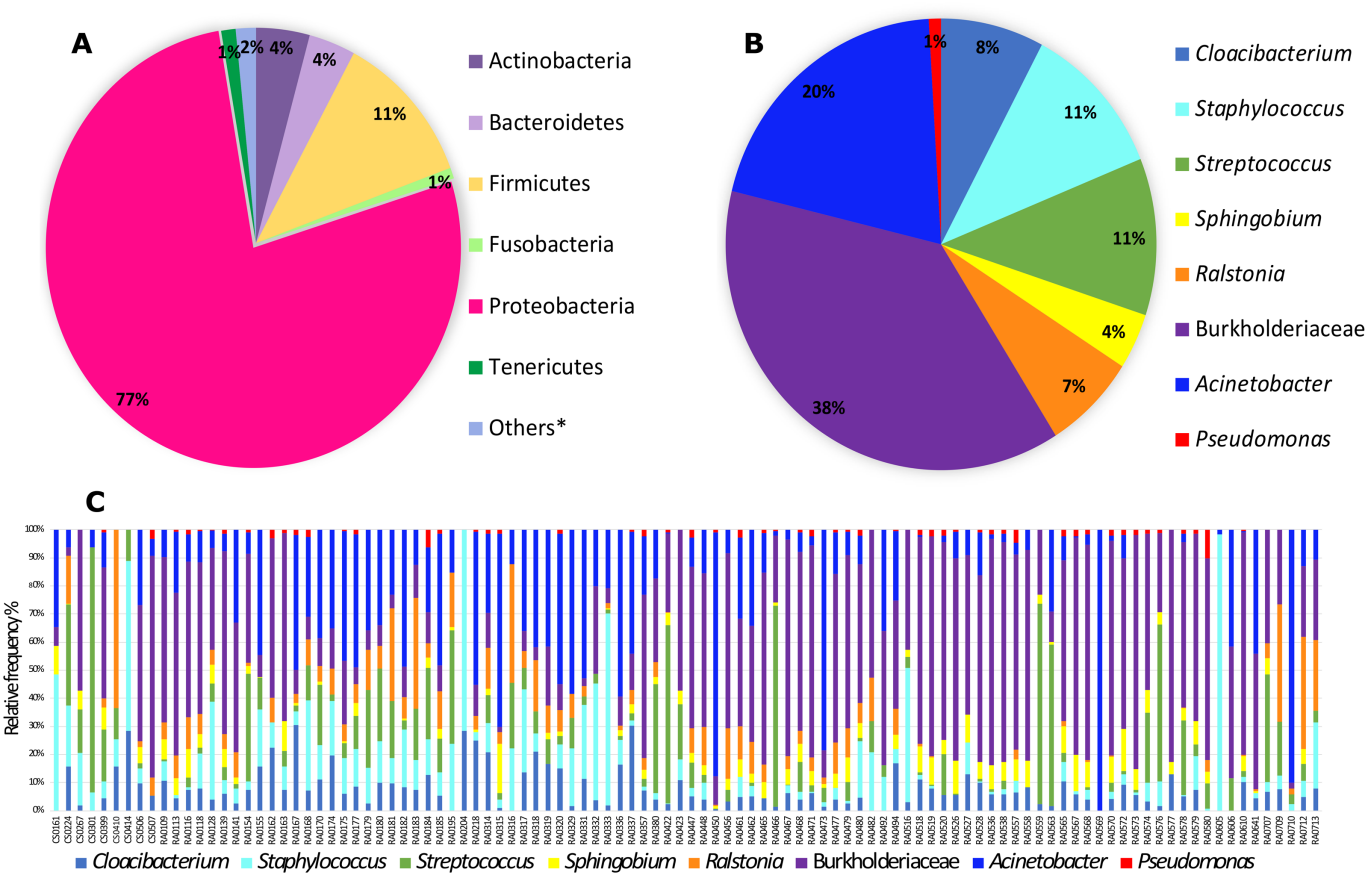
Relative frequency of bacteria (phyla and genera) found in *Pheidole* samples. (A) Abundance of bacteria showing all phyla with more than 1% relative frequency in all samples. Phyla encompassing less than 1% relative abundance are called others and are listed in Table S2. (B) Frequency of ‘core’ microbiota (present in at least 50% of samples) of genera (when available) found in samples of *Pheidole*. (C) Frequency of ‘core’ bacterial genera for each sample individually.

From the 118 samples initially considered for this study, we processed 106 samples that had the required amount of DNA and amplified PCR products for Illumina 16S rRNA gene sequencing plus two control sample. After demultiplexing we obtained a total of 11,349,500 sequence reads with an average length of 300 bp. These data were then subjected to DADA2 for denoising and resulted in a total of 5,645,544 sequences and 7,990 features. The DADA2 output was analyzed in Decontam in order to eliminate the contaminants based on a prevalence method and after this step we ended up with a final table that was also filtered in QIIME2 for ASVs with low total abundance and taxonomic filtering to eliminate any chloroplast, eukaryote or mitochondria resulting in 5,036,202 sequences and 1,787 features. This resulting table was then used in subsequent analyses. In total, we identified six bacterial phyla with more than 1% total relative frequency with Proteobacteria (77%) the major phylum, followed by Firmicutes (11%) and others with less than 5% relative frequency (Table S2, Fig. 1A). Our full feature table resulted in 611 ASVs (Table S3). The predominant ‘core’ bacteria in *Pheidole* (those found in >50% of the samples) is composed of 14 ASVs the most prevalent of which are in the family Burkholderiaceae (with 38% of total relative frequency), followed by the genus *Acinetobacter* (20%), *Streptococcus* and *Staphylococcus* (both with 11%), *Cloacibacterium* (8%) and *Ralstonia* (7%) and others with less than 5% frequency (Table S4 and Figs. 1B and 1C). Individual differences for each sample are illustrated in Fig. 1C. *Wolbachia* was found in less than 50% of the samples and to visualize its prevalence we analyzed the ‘core’ for bacteria found in >40% of the samples and we were able to identify *Wolbachia* as the most prevalent in this ‘core’ with a overall relative frequency of 26% (Table S5 and Fig. S1).

### Bacterial quantification

qPCR data showed that the estimated bacterial abundance in *Pheidole* ants does not vary when we compare the different ant samples that used different food resources and are from different locations. When we compare all the three categories (seed harvester, no seed harvesting and unknown food resource) the ANOVA showed no statistical significance (*p* = 0.724, *F* value = 0.342) between categories. We also found no differences for the Welch two sample *t*-test for estimated bacterial abundance when comparing only seed harvester and no seed harvesting ants (*p* = 0.461) and New World and Old World ants (*p* = 0.631) (Fig. S2 and Table S6).

### Alpha diversity

We found no differences (*p* > 0.05) in Shannon‘s diversity index with Kruskal Wallis pairwise comparison for all the categories we grouped our samples (food resource and geography by region and Old World x New World samples) suggesting that the community richness and equitability does not vary between those categories (Table S7). The number of observed features (qualitative measure of community richness) ranged from 4–153 and no difference was found between the categories we grouped our samples (*p* > 0.05) (Table S8). Faith’s Phylogenetic Diversity index ranged from 0.825–9.987 and we found no differences between the categories we grouped our samples (*p* > 0.05) suggesting that in a qualitative measure the community richness (that incorporates phylogenetic relationships between the features) also does not differ (Table S9).

If we examine alpha diversity for the ‘core’ microbiota we did find differences (*p* = 0.031, *q* = 0.092) in the Shannon‘s diversity index with Kruskal Wallis pairwise comparison for the category food resource suggesting that the diversity vary between seed harvesting and no seed harvesting ants with seed harvesting showing a higher index (Table S7). A correlation was also found for fine scale geography with differences between Australian and Neotropical (*p* = 0.016, *q* = 0.079) but we have to be careful interpreting this result because we have a small number of samples for Australian (*n* = 3). Differences for ‘core’ was also found between Nearctic and Neotropical samples (*p* = 0.0005, *q* = 0.006). For both of the fine scale differences we detected the Neotropical group has higher Shannon’s diversity index (Table S7). No difference was found between Old World and New World samples (Table S8). The number of observed features (qualitative measure of community richness) ranged from 7–15 and differences were detected in fine scale geography between Neotropical and Nearctic samples (*p* = 0.038, *q* = 0.325). Although we obtained uncorrected *P* values <0.05, it is important to highlight that some of the corresponding Q values exceed this threshold, except for Neartic and Neotropical differences. No differences were found in food resource and large-scale geography (or Old World and New World samples) (*p* > 0.05) (Table S8). Faith’s Phylogenetic Diversity index ranged from 1.58–2.01 and we found no differences between the categories we grouped our samples (*p* > 0.05) suggesting that in a qualitative measure the community richness (that incorporates phylogenetic relationships between the features) does not differ (Table S9).

Overall, these alpha diversity results highlight that in the general microbiota of *Pheidole* there was high diversity of bacteria found in our samples but there were no signatures of differences in the community richness in our samples and between the categories analyzed. But when we look at the ‘core’ microbiota the data suggest that the community richness might vary between seed harvesting and no seed harvesting ants and between some fine scale geographic ranges of our sample.

### Beta-diversity

The bacterial community composition did differ among our samples as shown in our beta diversity analysis. When comparing the data from all samples (i.e., not divided by groups in the categories analyzed) we did find significance only in the weighted larger scale region (*p* = 0.05) and unweighted fine scale (*p* = 0.032) (Table 1). But if we look at the comparison between the pairwise groups within each category we divided our samples (i.e., in food resource we compared the seed, no seed and no info group, in region between all the seven regions, and between Old World and New World), we find signals that the bacterial community composition is distinct especially in our not core filtered table. We find significant differences among unweighted Unifrac distances (qualitative measure that include phylogenetic relationships between features) for the category different food resource between seed harvester and no seed harvester ants (*p* = 0.05, *q* = 0.165) and weighted Unifrac distances (quantitative measure of community dissimilarity that includes phylogenetic relationships between features) for categories which include fine scale region and large scale as New World and Old World (*p* < 0.05, *q* = 0.189 –0.441) at the 611 ASVs from our full feature table not ‘core’ filtered (Table 2). But the beta diversity analysis of ‘core’ ASVs showed that the community composition did not differ for food resource but only differ between Neotropical and Australian groups (*p* = 0.05, *q* = 0.53) (Table S10 for all the results). Again, although we obtained uncorrected *P* values <0.05 for these results, it is important to highlight that the corresponding Q values exceed this threshold.

**Table 1:** PERMANOVA results from beta diversity analysis from general ASV table and core ASV table (bacteria found in at least 50% of samples). The significant values are highlighted in bold. Permutations = 999.

	Weighted		Unweighted	
	*p*-value	Pseudo-F	*p*-value	Pseudo-F
**General ASV table**				
Food resource	0.221	1.314	0.205	1.129
Region (fine scale^*^)	0.206	1.259	**0.032**	1.245
Region (larger scale^**^)	**0.05**	1.743	0.215	1.161
**Core (50%) ASV table**				
Food resource	0.443	0.930	0.709	0.377
Region (fine scale^*^)	0.697	0.728	0.883	0.386
Region (larger scale^**^)	0.894	0.156	0.976	−0.301

**Notes.**

* Fine scale is related to samples that came from different regions of the globe which includes Afrotropical, Australian, Indomalaya, Nearctic, Neotropical, Oceania and Paleartic.

** Larger scale is related to samples that came from Old World or New World collection.

**Table 2:** Pairwise PERMANOVA results from beta diversity analysis from general ASV table for each pair inside each category. The significant values found for each group inside each category are highlighted in bold. Permutations = 999.

		Weighted		Unweighted	
		*p*-value	*q*-value	*p*-value	*q*-value
Food resource	no info × no seed	0.112	0.336	0.488	0.488
	no info × seed	0.299	0.4485	0.261	0.3915
	no seed × seed	0.492	0.492	**0.055**	**0.165**
Region fine scale	Afrotropical × Australian	0.546	0.764	0.668	0.714
	Afrotropical × Indomalaya	0.348	0.764	0.478	0.6986
	Afrotropical × Nearctic	0.777	0.859	0.595	0.714
	Afrotropical × Neotropical	0.461	0.764	**0.03**	**0.21**
	Afrotropical × Oceania	0.726	0.847	0.075	0.286
	Afrotropical × Palearctic	0.608	0.798	0.534	0.701
	Australian × Indomalaya	0.235	0.764	0.401	0.699
	Australian × Nearctic	0.326	0.764	0.63	0.714
	Australian × Neotropical	0.255	0.764	**0.049**	**0.257**
	Australian × Oceania	0.517	0.764	0.101	0.286
	Australian × Palearctic	0.385	0.764	0.499	0.699
	Indomalaya × Nearctic	**0.036**	**0.441**	0.385	0.699
	Indomalaya × Neotropical	**0.042**	**0.441**	0.477	0.699
	Indomalaya × Oceania	0.659	0.814	0.109	0.286
	Indomalaya × Palearctic	0.913	0.959	0.837	0.837
	Nearctic × Neotropical	0.233	0.764	**0.009**	**0.189**
	Nearctic × Oceania	0.453	0.764	0.091	0.286
	Nearctic × Palearctic	0.308	0.764	0.496	0.699
	Neotropical × Oceania	0.524	0.764	**0.03**	**0.21**
	Neotropical × Palearctic	0.227	0.764	0.354	0.699
	Oceania × Palearctic	1.0	1.0	0.68	0.714
Region larger scale	Old World × New World	**0.036**	**0.036**	0.214	0.214

These beta diversity results were also investigated with an ANCOM analyses in order to see the differentially abundant taxa in our sample groups. We found that the taxa responsible for the differences in beta diversity for the general data (not ‘core’ filtered) for the fine scale region category are the genera *Blastococcus* (*W* = 607), *Prevotella* (*W* = 661), *Truepera* (*W* = 613), *Nosocomiicoccus* (*W* = 648), *Rickettsiella* (*W* = 615), *Psychrobacter* (*W* = 645) and Planococcaceae family (*W* = 664). For different food resource although results from the beta diversity indicate that there is a qualitative difference in bacterial community among seed harvester and no seed harvester, ANCOM analyses did not depicted any particular taxa that would be responsible for the differential beta diversity. For the ‘core’ data the taxa responsible for the significant difference between Neotropical and Australian groups are the genera *Sphingobium*, *Ralstonia* and unidentified members of the Burkholderiaceae family.

**Figure 2:**
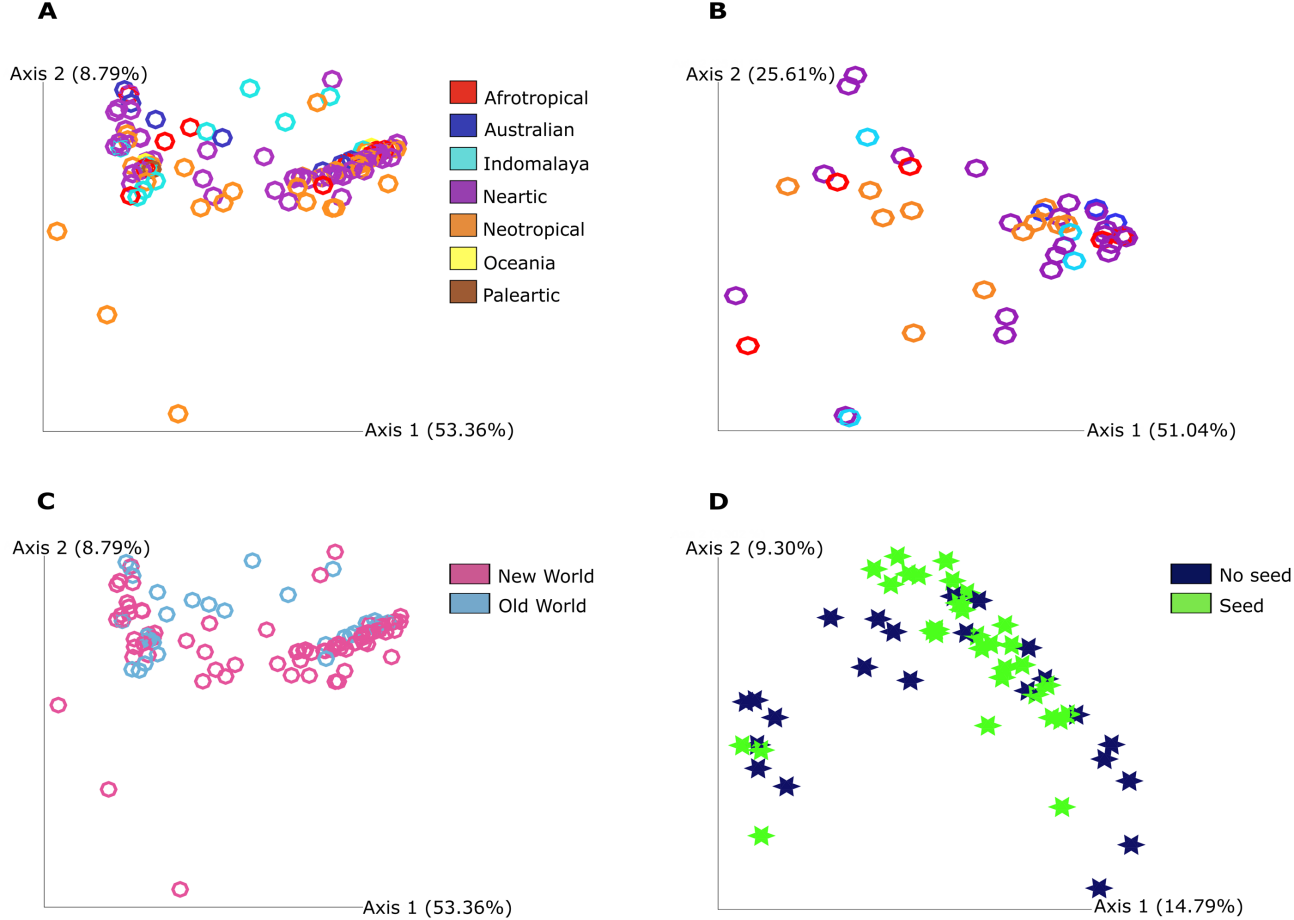
Principal Coordinate Analysis (PCoA) plot of *Pheidole* bacterial communities at 7,000 sampling read depth. (A) Weighted Unifrac metric of the samples from different regions from not ‘core’ filtered table. The colors indicate all the different groupings in this category: Afrotropical, Australian, Indomalaya, Nearctic, Neotropical, Oceania and Palearctic. (B) Weighted Unifrac metric of the samples from Old World and New World from not ‘core’ filtered table. The different colors indicate the two different groupings in this category: Old World and New World ants. (C) Weighted Unifrac metric of samples from different regions from ‘core’ table (50%). The colors indicate all the different groupings in this category: Afrotropical, Australian, Indomalaya, Nearctic and Neotropical. (D) Unweighted Unifrac metric of samples from different food resources. The different colors described in the legend indicate the two different groupings that were found to be distinct: no seed harvesting ants and seed harvesting ants. The significance was obtained by PERMANOVA pairwise test and we illustrate here only the groups that show significance in this test.

The PCoA plot shows that the grouping between the categories we found significance in our statistical analysis is not strong in a graphical view of the ordination. This highlights that the bacterial communities do differ as statistical analysis show but in a discrete way since no clear grouping was found in this visualization and mostly in qualitative measure that include phylogenetic relationships between features for food resource and quantitative measure of community dissimilarity that includes phylogenetic relationships between features for geography (Fig. 2).

### Heatmap

**Figure 3:**
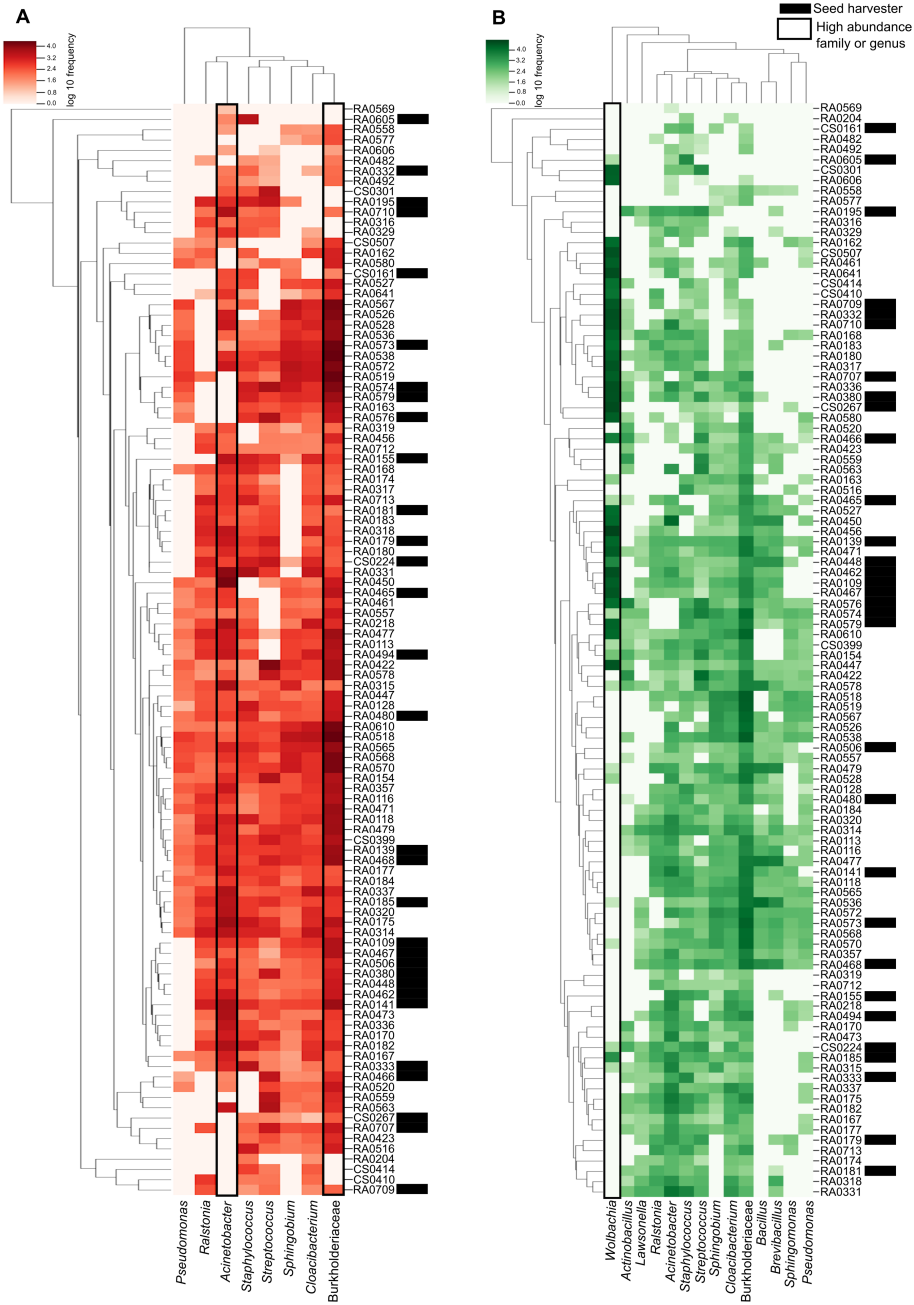
Heatmap representation of *Pheidole* ‘core’ microbiome showing the frequency of each ASV. The ASVs were analyzed at the genus level (when this classification is available) and are indicated on the bottom and the samples are indicated in the left. Both were organized by the Bray–Curtis method to generate a hierarchical clustering of the bacterial communities and the samples. Darker red or green indicate higher abundance of the ASV and the lighter red or green smaller abundance. (A) Heatmap representation of *Pheidole* ‘core’ microbiome showing the frequency of each ASV present in >50% of the samples. We can clearly see the high abundance of Burkhoderiaceae family and *Cloacibacterium* genus. Seed harvester samples are indicated with an asterisk. (B) Heatmap representation of *Pheidole* ‘core’ microbiome showing the frequency of each ASV present in >40% of the samples. We can clearly see the high abundance of *Wolbachia*.

The heatmap shows the relationship between our samples and each ASV with the darker color scale corresponding to more abundant bacteria found in the microbiome data. ASVs and samples sorted by dendrograms show how related are the ASVs and the samples according with the phylogenetic distance and ASV shared respectively (Fig. 3). We can see that in the ‘core’ (>50%) the high abundance of unidentified members of the Burkholderiaceae and the genus *Cloacibacterium* (the two most prevalent in our samples) and its distribution in almost all samples (Fig. 3A). We highlight that in ‘core’ for bacteria found in >40% of the samples, *Wolbachia* is the most abundant illustrated in darker green in Fig. 3B.

### Mantel

To understand if the phylogeny and the fine scale geography are influencing the bacterial communities in *Pheidole* we also ran Mantel tests. For the Mantel tests we found a correlation between host geographic location and bacterial community (*p* = 0.042, Spearman rho = 0.0809595) when looking at the total bacterial community (not core filtered) using the weighted Unifrac distance matrix, giving support to the results above indicating that there were correlations between the bacterial community with the geographical origin of the host. We did not find a correlation with host phylogeny and bacterial community suggesting that phylogeny is not driving *Pheidole’s* microbiota.

## Discussion

This is the first study to investigate the microbiota of *Pheidole* ants across a diverse collection of species from several geographical locations and encompassing numerous seed harvesting species. Although determining why some lineages are more diverse is often difficult, we wanted to study the microbial community associated with *Pheidole* ants to understand what forces shape the variation in their bacterial community and if this could account for their diet shifts. It was already known from several studies exploring ant microbial composition that the forces influencing the bacterial community in ants are diverse ranging from diet, geography, species and phylogeny (Russell et al., 2009b; Anderson et al., 2012; Hu et al., 2014; Sanders et al., 2017a; Sanders et al., 2017b; Hu et al., 2017; Hu et al., 2018; Sanders et al., 2014; Lanan et al., 2016; Moreau & Rubin, 2017; Ramalho, Bueno & Moreau, 2017b; Ramalho, Bueno & Moreau, 2017a; Vieira et al., 2017). Our data show that in *Pheidole* ants several factors have discrete influence on the microbiome and the bacterial community is relatively diverse as indicated by the alpha and beta diversity analyses. We found moderately high diversity of bacteria in our alpha diversity analysis and we found that the ‘core’ microbiota might differ for the categories of food resource and fine scale geography suggesting that bacterial relative abundance does vary between seed harvesting and no seed harvesting ants and some geographical locations. Beta diversity analysis shows that there is a difference in the bacterial community in *Pheidole* samples when we compare different categories, suggesting that geographic location may influence the general bacterial community in a quantitative way (abundance) and food resource may influence the general bacterial community in qualitative way (presence or absence). Our data also show that *Pheidole* harbors a core microbiome suggesting a possible general function of this microbial community for *Pheidole* ants and the Mantel test suggests that *Pheidole* bacterial diversity is partially explained by the geography of the host.

### Main bacteria associated with *Pheidole*

*Pheidole* microbial community was dominated by the phyla Proteobacteria and Firmicutes corroborating previous studies from the gut of several insects (Colman, Toolson & Takacs-Vesbach, 2012; Esposti & Romero, 2017; Jones, Sanchez & Fierer, 2013; Yun et al., 2014) including ants (Brown & Wernegreen, 2016). As in these previous studies the most prevalent bacteria in our samples were those already found associated with insect and ant guts suggesting the important and dominant presence of these bacterial in *Pheidole* microbiota.

At the family level the most prevalent ‘core’ bacteria was an unidentified Burkholderiaceae. We emphasize that for ASV designation it is not always possible to have bacterial identification at the species level since we cannot always match samples in the databases at the genus or species taxonomic level. In the absence of a close match, the assignment is made at the previous taxonomic level (i.e., genus, family, etc.). This group of bacteria has a wide diversity of taxa and without an exact match to our sample it would be only speculative take any further discussion on this topic. Nevertheless, it is important to highlight that Burkholderiaceae has been documented associated with several ant species (Van Borm et al., 2002; Russell et al., 2009b; He et al., 2011; Kautz, Rubin & Moreau, 2013; Lindström et al., 2018) and in all beetle species analyzed by Montagna et al. (2014). The Burkholderiales order was documented in *Cephalotes* ants and linked with a capacity to convert uric acid into urea (Hu et al., 2018). And *Burkholderia* sp. was already documented in leaf-cutting ants (*Atta sexdens rubropilosa*) and associated with antibiotic production (Santos et al., 2004).

The second most prevalent was the genus *Acinetobacter*, with 20% relative frequency, which was already documented as common in arthropod microbiota (Esposti & Romero, 2017), in *Solenopsis* ant species (Ishak et al., 2011), in *Nasonia* (Brucker & Bordenstein, 2012), wasps (Paulson, Von Aderkas & Perlman, 2014) and present in the microbiota of *Atta* fungus-growing ants (Meirelles et al., 2016).

*Streptococcus* and *Staphylococcus* were the third most prevalent, both with 11% relative frequency. Both comprise genera that are commonly found in the ambient or associated with human and animal, but are not uncommon in insects. *Streptococcus* has already been documented as insect pathogens (Bulla, Rhodes & St. Julian, 1975) and is present in the microbiome of several termite species (Eutick, O’Brien & Slaytor, 1978) and bed bugs (Meriweather et al., 2013). *Staphylococcus* has been detected in guts of adult workers of *Acromyrmex echinatior* ants (Zhukova et al., 2017), moth and beetle (Ignasiak & Maxwell, 2017) and termite species (Eutick, O’Brien & Slaytor, 1978).

*Cloacibacterium* was the fourth most prevalent with 8% relative frequency. Although not very common in insect microbiomes it has been observed in the fungus gardening ant *Atta texana* (Buckley, 1860) (Meirelles et al., 2016) and also bed bugs (Meriweather et al., 2013).

The fifth most prevalent bacterial genus found in the core from our *Pheidole* samples was *Ralstonia* (7%), with its presence already documented in guts of *Atta* fungus gardening ants (Zhukova et al., 2017), the microbiome of arthropods in general (Esposti & Romero, 2017), in all life stages of *Bactericera cockerelli* psyllids (Hail, Dowd & Bextine, 2012) and in carabid beetles that consume seeds (Lundgren & Lehman, 2010). This genus has also been documented as an important and abundant member in the wasp microbiome (Paulson, Von Aderkas & Perlman, 2014) and also in the gut of *Illeis koebelei* ladybird beetles (Yun et al., 2014). Paulson, Von Aderkas & Perlman (2014) speculated that this bacterial genus could play an important role in nutrient recycling in *Megastigmus* wasps - which infest seeds to undergo their development - and latter documented that in the *Megastigmus* transcriptome the majority of bacterial annotation was represented by sequences from *Ralstonia* with highly expressed mobile elements (Paulson et al., 2016). It is interesting that *Pheidole* ants, the *Megastigmus* wasp and *Harpalus pensylvanicus* carabids, all with seed related lifestyles, harbor high abundance of *Ralstonia* and future studies focusing on the function or even location within the host are necessary to better understand these results.

It is well-known that *Wolbachia* is one of the major endosymbionts of ants but relatively little is known about its function (Russell et al., 2009a; Russell et al., 2009b; Russell, Sanders & Moreau, 2017; Pontieri et al., 2017), and its distribution across different ant species. Our results highlight that in *Pheidole* this endosymbiont is present in relatively high frequency (45.3% of all samples from this study; Table S5). It is important to highlight that for the ‘core’ microbiota present in >50% of the samples, *Wolbachia* was not present but when the ‘core’ is relaxed to >40% of the samples *Wolbachia* is assigned as the most prevalent (26%). Our results corroborate previous findings from Russell et al. (2009a) describing *Wolbachia* in *Pheidole* ants in several species analyzed. From 82 sampled ants from genus *Pheidole* from that former study the authors did find *Wolbachia* infection in 27 samples (33% of the sample infected). Here we found that in *Pheidole*, *Wolbachia* has high relative frequency (Fig. 3 and Fig. S1).

### Geographic and Phylogenetic influence in bacterial community

Our data show that the *Pheidole* bacterial community is distinct between geographical location with differentially abundant taxa responsible for the differences (i.e., *Blastococcus*, *Prevotella*, *Truepera*, *Nosocomiicoccus*, *Rickettsiella*, *Psychrobacter,* Planococcaceae family, *Sphingobium*, *Ralstonia* and Burkholderiaceae family). Alpha and beta diversity and also Mantel tests corroborate these findings. But these differences are not obvious in the PCoA analysis likely because the differences in the bacterial communities are not large. Mantel tests supports the results from the beta diversity analysis with geographic distance explaining ASV diversity in our *Pheidole* samples but only for the quantitative measure of community divergence that incorporates phylogenetic relationships (weighted Unifrac).

Host microbiota correlating with geographic location has already been documented in other animals (Lee, Husseneder & Hooper-Bùi, 2008; Linnenbrink et al., 2013; Welch, Macias & Bextine, 2015; Bird et al., 2018) highlighting that the host geographic location may influence the associated microbes in some animal groups and emphasizing that studies including samples from across the host’s distribution are necessary to understand the evolutionary forces shaping microbiota. In larvae of fourth-instar *Solenopsis invicta* ants Lee and coworkers found that the bacterial community differed in each sampled location suggesting that this may be explained by the transient organisms native to the soil from each location (Lee, Husseneder & Hooper-Bùi, 2008). Apart from being already documented in other insect species, some bacteria found in our *Pheidole* samples have also been reported in the soil and other environments (Esposti & Romero, 2017). While our data suggest that these bacteria may be the result of transient environmental contaminants it is also important to remember that that geographic congruence can also be influenced by temperature, food availability and soil composition and only further work can confirm the extent these bacteria are picked up in the environment in influencing the microbiome of those ants.

In *Pheidole* ants*,* host phylogeny does not influence bacterial diversity with results from our analysis from the Mantel test and the presence of a ‘core’ microbiota supporting this finding. Previous study on other ant groups have found the opposite scenario. It has been documented for *Cephalotes* turtle ants (Sanders et al., 2014) and also for *Polyrhachis* spiny ants (Ramalho, Bueno & Moreau, 2017a) that host phylogeny influences bacterial diversity in these groups. These incongruency with our findings for *Pheidole* highlights the fact that each group has its own evolutionary path and evolutionary forces shaping microbiota and it seems that congruency with phylogeny and microbiota is not a trend for all groups of ants.

### Microbiota is not completely related to food preferences

Seed milling and harvesting is considered one potential key innovation that has evolved multiple times and has been lost in some lineages in *Pheidole* ants (Moreau, 2008). The question remains whether symbiotic microbes may have permitted this group of ants to leverage this novel food source. Our qPCR data show that the bacteria quantity in *Pheidole* does not differ related to feeding habit (seed and no seed). However, if we look at the diversity of the bacterial community, we find support of a divergent bacterial community between seed harvesting and no seed harvesting species. These results highlight that there might be an association of bacterial community in ants related to their feeding habit, but not substantially because the same bacteria were found in all *Pheidole* samples analyzed and the differences are minor since no significant groupings were shown in our graphical data and ANCOM analysis between seed harvesters and no seed harvesters.

Our finding suggests that specific bacteria are likely not required in the evolution of novel food sources in *Pheidole* ants. However the recovered bacteria still could play a role in seed harvesting. It is important to note that our study focused in the whole ant microbiome and environmentally-acquired and also vertically transmitted bacteria could blur the seed harvesting effect in our samples (Jones, Sanchez & Fierer, 2013) and since we find geographical congruence in our data we cannot rule out the possibility of such acquisition of microbes from the surrounding environment in our samples. New studies should be designed and complement our results for this group of ants to completely exclude external factors that could influence the results such as environmental acquired and food retained in the digestive tract.

## Conclusion

In this study, we sequenced and identified the microbiome of over 100 species of the hyper-diverse ant genus *Pheidole*. These samples cover the worldwide geographic range of the genus and include species that harvest and eat seeds to determine if microbial associations may be explained by their geographic location or diet. We found that *Pheidole* harbors a stable core microbiome. Furthermore, we found evidence that geography and a seed-harvesting diet might shape the diversity of the bacterial communities, although none of them alone explains the bacterial diversity in this ecologically important and evolutionarily diverse group of insects.

##  Supplemental Information

10.7717/peerj.8492/supp-1 Figure S1 (A) Frequency of ‘core’ bacterial genera for each sample and (B) for all samples individually

10.7717/peerj.8492/supp-2 Figure S2 Box plot showing the log10 transformed data of median, smallest and largest values, 25% and 75% quartiles and outliers (A) Box plot from transformed bacteria quantities from *Pheidole* ants organized by food resource used. (B) Box plot from transformed bacteria quantities from *Pheidole* organized by Old World and New World ant collection site.

10.7717/peerj.8492/supp-3 Table S1 Samples used in this study Sample ID, genus and species, region, continent, country of collection and food resource.

10.7717/peerj.8492/supp-4 Table S2 Relative frequency for phyla level from general bacteria (not core filtered) found in Pheidole ants Bacteria phylum called as others are the sum of all the listed bacteria bellow. Those correspond to the bacteria with less than 1% relative frequency each.

10.7717/peerj.8492/supp-5 Table S3 General ASV table at genus level This is the resulting table after Decontam filtering and posterior filtering.

10.7717/peerj.8492/supp-6 Table S4 Relative frequencies for ‘core’ microbiota Table show ASVs found associated with Pheidole samples (bacteria found in >50% of our samples) at genera level (when available).

10.7717/peerj.8492/supp-7 Table S5 Relative frequency of ‘core’ microbiota Table show ASVs found associated with Pheidole samples (bacteria found in >40% of our samples) at genera level (when available).

10.7717/peerj.8492/supp-8 Table S6 qPCR data averaged, standard deviation and log10 Food resource used based on Moreau (2008) and location (New ×Old World) are indicated for each sample.

10.7717/peerj.8492/supp-9 Table S7 Shannon index for each sample and category used and for ‘core’ microbiota and Kruskal Wallis pairwise data for each sample and for ‘core’ S7.1: Kruskal Wallis pairwise data for sample grouped for food resource. S7.2: regions. S7.3: Old World and New World samples. S7.4: Shannon index for each sample and categorical used from ‘core’ microbiota. S7.5: Kruskal Wallis pairwise data for sample grouped for food resource from ‘core’ microbiota. S7.6: regions. S7.7: Old World and New World samples.

10.7717/peerj.8492/supp-10 Table S8 Number of observed OTU for each sample and for ‘core’ microbiota data and Kruskal Wallis pairwise data for each category and for ‘core’ S8.1: Kruskal Wallis pairwise data for sample grouped for food resource. S8.2: regions. S8.3: Old World and New World. S8.4: Number of observed OTU for each sample for ‘core’ microbiota. S8.5: Kruskal Wallis pairwise data for sample grouped for food resource for ‘core’ microbiota. S8.6: regions. S8.7: Old World and New World samples.

10.7717/peerj.8492/supp-11 Table S9 Faith’s Phylogenetic Diversity for all sample and for ‘core’ for each categories analyzed Kruskal Wallis pairwise data for sample grouped for food resource. S9.2: regions. S9.3: Old World and New World samples. S9.4: Faith’s Phylogenetic Diversity for all sample and categories analyzed for ‘core’. S9.5: Kruskal Wallis pairwise data for sample grouped for food resource. S9.6: regions. S9.7: Old World and New World category.

10.7717/peerj.8492/supp-12 Table 10 Permanova-pairwise results from core filtered table for weighted unifrac Permanova-pairwise results from core filtered table for unweighted unifrac for type of food resource: seed, no seed and no info (which represents the samples that we don’t have information of the kind of food resource used).
Permanova-pairwise results from core filtered table for weighted unifrac for type of food resource: seed, no seed and no info (which represents the samples that we don’t have information of the kind of food resource used).
Permanova-pairwise results from core filtered table for unweighted unifrac for region of ant collection.
Permanova-pairwise results from core filtered table for weighted unifrac for region of ant collection. Highlighted in gray are statistical significant data.
Permanova-pairwise results from core filtered table for unweighted unifrac for Old World and New World ants. Highlighted in gray are statistical significant data.
Permanova-pairwise results from core filtered table for weighted unifrac for Old World and New World ants. Highlighted in gray are statistical significant data.

## Additional Information and Declarations

### Data Availability

The following information was supplied regarding data availability: All sequence data is available in NCBI SRA: SRP156872.
