# Supplementary figures and images for: Influence of host phylogeny, geographical location and seed harvesting diet on the bacterial community of globally distributed Pheidole ants
Source: PeerJ. 2020 Feb 4;8:e8492. doi: 10.7717/peerj.8492 (PMC7006521; doi:10.7717/peerj.8492)

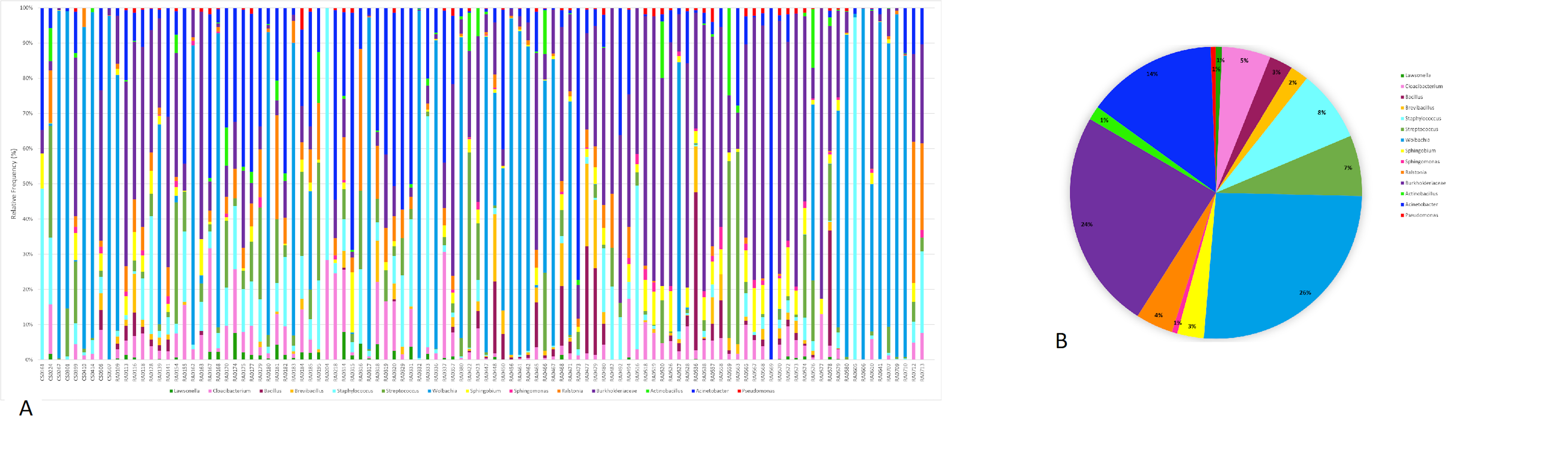

Supplement: Figure S1 [file peerj-08-8492-s001.png]

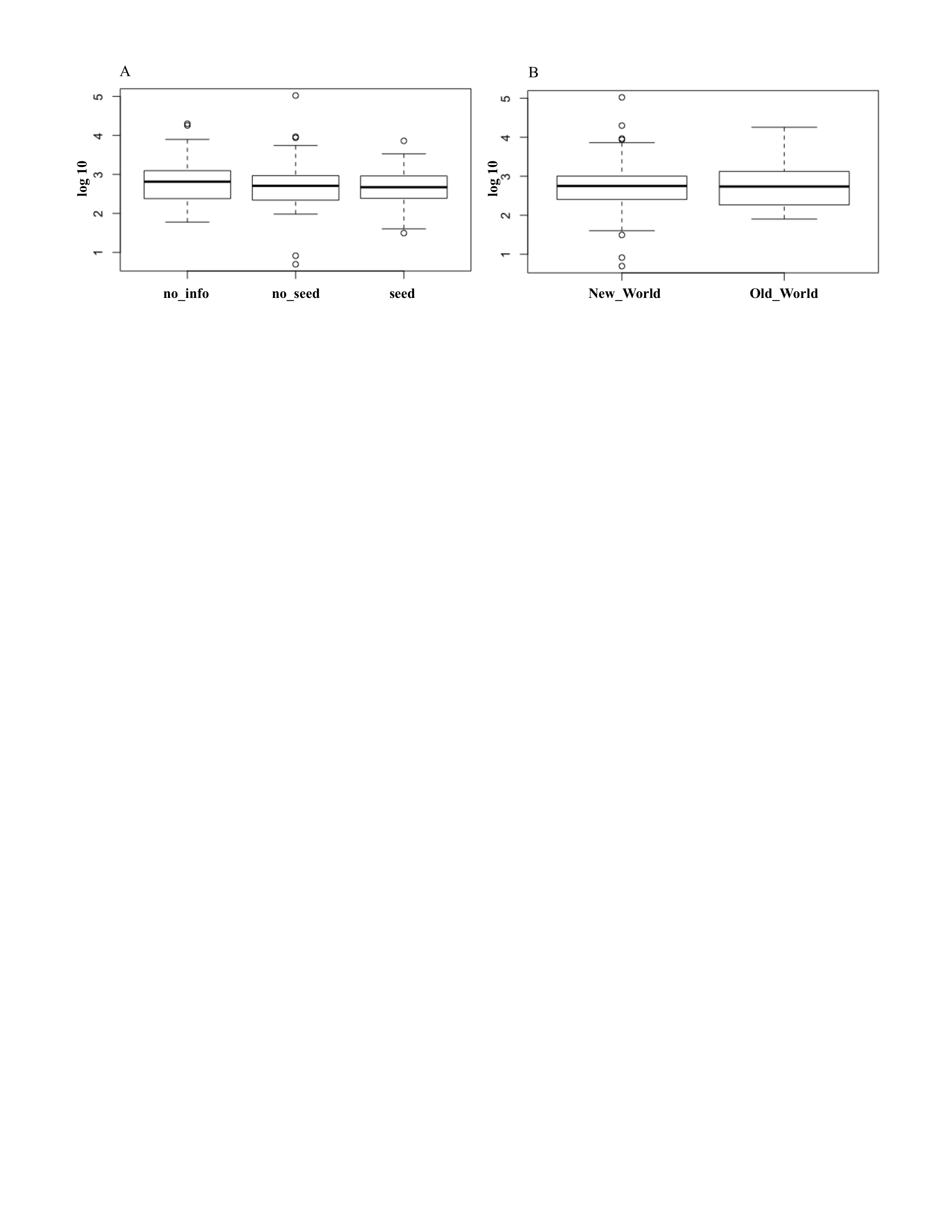

Supplement: Figure S2 — (A) Box plot from transformed bacteria quantities from Pheidole ants organized by food resource used. (B) Box plot from transformed bacteria quantities from Pheidole organized by Old World and New World ant collection site. [file peerj-08-8492-s002.png]
